# Establishment of a clinical SPECT/CT protocol for imaging of ^161^Tb

**DOI:** 10.1186/s40658-020-00314-x

**Published:** 2020-07-01

**Authors:** I. Marin, T. Rydèn, M. Van Essen, J. Svensson, N. Gracheva, U. Köster, J. R. Zeevaart, N. P. van der Meulen, C. Müller, P. Bernhardt

**Affiliations:** 1 Department of Radiation Physics, Institution of Clinical Science, Sahlgrenska Academy, University of Gothenburg, Gothenburg, Sweden; 2 Department of Medical Physics and Bioengineering, Sahlgrenska University Hospital, Gula Stråket 2B, 413 45 Gothenburg, Sweden; 3 Department of Clinical Physiology, Sahlgrenska University Hospital, Gothenburg, Sweden; 4 Department of Oncology, Institution of Clinical Science, Sahlgrenska Academy, University of Gothenburg, Gothenburg, Sweden; 5 Center for Radiopharmaceutical Sciences ETH-PSI-USZ, Paul Scherrer Institute, Villigen-PSI, Villigen, Switzerland; 6 Institute Laue-Langevin, Grenoble, France; 7 Radiochemistry, South African Nuclear Energy Corporation (Necsa), Brits, South Africa; 8 Laboratory of Radiochemistry, Paul Scherrer Institute, Villigen-PSI, Villigen, Switzerland

**Keywords:** ^161^Tb, SPECT, Image quality, Targeted radionuclide therapy

## Abstract

**Background:**

It has been proposed, and preclinically demonstrated, that ^161^Tb is a better alternative to ^177^Lu for the treatment of small prostate cancer lesions due to its high emission of low-energy electrons. ^161^Tb also emits photons suitable for single-photon emission computed tomography (SPECT) imaging. This study aims to establish a SPECT protocol for ^161^Tb imaging in the clinic.

**Materials and methods:**

Optimal settings using various γ-camera collimators and energy windows were explored by imaging a Jaszczak phantom, including hollow-sphere inserts, filled with ^161^Tb. The collimators examined were extended low-energy general purpose (ELEGP), medium-energy general purpose (MEGP), and low-energy high resolution (LEHR), respectively. In addition, three ordered subset expectation maximization (OSEM) algorithms were investigated: attenuation-corrected OSEM (A-OSEM); attenuation and dual- or triple-energy window scatter-corrected OSEM (AS-OSEM); and attenuation, scatter, and collimator-detector response-corrected OSEM (ASC-OSEM), where the latter utilized Monte Carlo-based reconstruction. Uniformity corrections, using intrinsic and extrinsic correction maps, were also investigated. Image quality was assessed by estimated recovery coefficients (RC), noise, and signal-to-noise ratio (SNR). Sensitivity was determined using a circular flat phantom.

**Results:**

The best RC and SNR were obtained at an energy window between 67.1 and 82.1 keV. Ring artifacts, caused by non-uniformity, were removed with extrinsic uniformity correction for the energy window between 67.1 and 82.1 keV, but not with intrinsic correction. Analyzing the lower energy window between 48.9 and 62.9 keV, the ring artifacts remained after uniformity corrections. The recovery was similar for the different collimators when using a specific OSEM reconstruction. Recovery and SNR were highest for ASC-OSEM, followed by AS-OSEM and A-OSEM. When using the optimized parameter setting, the resolution of ^161^Tb was higher than for ^177^Lu (8.4 ± 0.7 vs. 10.4 ± 0.6 mm, respectively). The sensitivities for ^161^Tb and ^177^Lu were 7.41 and 8.46 cps/MBq, respectively.

**Conclusion:**

SPECT with high resolution is feasible with ^161^Tb; however, extrinsic uniformity correction is recommended to avoid ring artifacts. The LEHR collimator was the best choice of the three tested to obtain a high-resolution image. Due to the complex emission spectrum of low-energy photons, window-based scatter correction had a minor impact on the image quality compared to using attenuation correction only. On the other hand, performing attenuation, scatter, and collimator-detector correction clearly improved image quality. Based on these data, SPECT-based dosimetry for ^161^Tb-labeled radiopharmaceuticals is feasible.

## Introduction

Targeted radionuclide therapy of neuroendocrine tumors using ^177^Lu-labeled somatostatin analogs has been performed for more than a decade [1]. The NETTER-1 Phase III study demonstrated that a standard protocol with four cycles of 7.4 GBq of the radionuclide was considered safe, with improved response rates compared to that of standard care [2]. The radiolanthanide luthetium-177 (^177^Lu) is a medium-energy electron emitter, having electron range suitable for treatment of small tumors. When treating micro-clusters of proliferating tumor cells, however, the absorbed energy fraction will be low and high absorbed doses may be challenging to achieve [3, 4].

Recently, several clinical studies using ^177^Lu, labeled to prostate-specific membrane antigen (PSMA), have shown encouraging results in the treatment of aggressive metastatic prostate cancer [5–8]. Complete remission measured by the prostate-specific antigen (PSA) biochemical marker, as well as positron emission tomography (PET) with gallium-68 (^68^Ga)-PSMA-11, has been reported. In a recent retrospective analysis, it was also demonstrated that this treatment modality is superior to established third-line treatments while also having fewer side effects [9].

Despite these encouraging results, relapses occur in these late-stage-treated prostate cancers, which may partly be due to the non-optimal range of the electrons emitted from ^177^Lu decay [10].

Recently, Haberkorn and co-workers utilized PSMA-617, labeled with the alpha emitter actinium-225 (^225^Ac), to ensure high absorbed doses to micro-metastases of prostate cancer [11, 12]. Impressive results were obtained in these patients with abundant diffused metastatic spread. Conclusions cannot be drawn nor comparisons made for the treatment efficacy of ^177^Lu versus ^225^Ac in these limited studies, but the results indicate that short-ranged radiation is of value in the treatment of these late-stage metastatic prostate cancer cases.

Dosimetric evaluation of radionuclides for the treatment of small tumors demonstrates that terbium-161 (^161^Tb) has a unique electron emission pattern for treatment of metastatic disease; preclinical studies have demonstrated its therapeutic potential [3, 13–15]. The recent development of its production has demonstrated that ^161^Tb can be produced as no-carrier-added product and in therapeutic quantities [13]. Preclinically, it was demonstrated that ^161^Tb-folate was more effective in the treatment of folate-receptor-positive tumors than its ^177^Lu counterpart, while there were no increased side effects on the kidneys [13, 16]. More recently, the superior effect of ^161^Tb over ^177^Lu was confirmed in a prostate cancer model using radiolabeled PSMA-617 [15].

The photon emissions from ^161^Tb enable imaging of the radionuclide using a γ-camera, including single-photon emission computed tomography/computed tomography (SPECT/CT). The energies of photons emitted by ^161^Tb are all in the low range (Table 1), however. Preclinically, it was shown that ^161^Tb can be imaged using a small-animal SPECT/CT scanner [15, 16]. The feasibility of imaging ^161^Tb with a clinical SPECT scanner has yet to be investigated and forms the focus of this study.

**Table 1:** Decay characteristics for ^161^Tb and ^177^Lu [17]. Photon emission with low yields (< 0.3%) have been omitted

	^161^Tb	^177^Lu
Half-life [days]	6.9	6.7
Mean energy β^-^ [keV]	154	134
Energy γ (yield [%])	25.7 (23)	112.9 (6.2)
	48.9 (17)	208.4 (10.4)
	57.2 (1.8)	
	74.6 (10)	
	87–550 (0.5)	
Energy X-ray (yield [%])	6.50 (6.8)	7.90 (1.5)
	7.20 (1.5)	9.02 (1.1)
	7.37 (2.4)	54.6 (1.6)
	7.64 (1.4)	55.8 (2.8)
	45.2 (6.6)	
	46.0 (11)	
	51.9 (1.2)	
	52.1 (2.3)	
Energy conversion and Auger electrons [keV] (yield [%])	0–0.1 (72)	0–0.1 (27)
	0.1–1 (738)	0.1–1 (55)
	1–10 (303)	1–10 (30)
	10–20 (42)	10–20
	20–30 (18)	20–30
	30–40 (39)	30–40
	0–40 (1213)	0–40 (111)

Clinical SPECT/CT image reconstructions are commonly performed using iterative algorithms, such as ordered subset expectation maximization (OSEM) algorithms [18]. Corrections, such as attenuation and scatter compensations, may be incorporated into the reconstruction algorithms. Attenuation-corrected OSEM (A-OSEM) ^177^Lu-DOTATATE SPECT has been used in several clinical dosimetry studies by Sandström et al. (e.g., [19]); however, most studies also include scattering corrections (AS-OSEM). In addition, the vendors of SPECT/CT equipment offer different model-based algorithms for collimator-detector corrections, e.g., Evolution, xSPECT, and Flash3D, resulting in improved image resolution. Such compensation for collimator-detector response has been applied in recent clinical dosimetry studies for ^177^Lu-DOTATATE and ^177^Lu-PSMA [20, 21]. Further image improvements may be achieved with Monte Carlo-based (MC) reconstruction techniques [22, 23]; which inherently incorporates compensation for attenuation, scatter, and detector-collimator response in the forward projection. While the simulation times have been considered to be too long for clinical implementation, we, nevertheless, recently implemented a fast MC-based reconstruction code that generated improved resolution compared to the in-depth resolution corrections method offered by the vendor of the SPECT/CT equipment [24]. This methodology was recently used for the evaluation of bone marrow dosimetry in ^177^Lu-DOTATATE treatments [25].

The aim of this study was to establish a SPECT/CT protocol for the measurement of ^161^Tb, where the challenge is the low-photon emission energy. The dependence of the image quality on the energy window settings was assessed. The SPECT/CT protocols were evaluated in phantom studies. Three different reconstruction methods were compared, namely, attenuation-corrected (AC) ordered subset expectation maximization (A-OSEM) reconstruction, attenuation- and scatter-corrected (SC) OSEM reconstruction (AS-OSEM), and AC, SC, and collimator-detector response (C)-corrected OSEM (ASC-OSEM), using MC-based reconstruction with the Sahlgrenska Academy Reconstruction code (SARec) [24].

## Materials and methods

### Camera and energy windows

All phantom measurements were performed using a Discovery NM/CT 670 Pro (GE Healthcare, Chicago, IL, USA) γ-camera. An energy spectrum was acquired for visualization of the photon emissions from ^161^Tb. The emission- and scatter-energy windows used are shown in Table 3. The lowest and highest emission windows were referred to as EM1 and EM2, respectively, while the scatter windows at the lowest and highest energy were referred to as SC1 and SC2, respectively. The center energies of EM1 and EM2 were equivalent to γ-emission energies of 48.9 and 74.6 keV, respectively.

### Collimators and reconstruction algorithms

The collimators used in this work include extended low-energy general purpose (ELEGP), medium-energy general purpose (MEGP), and low-energy high resolution (LEHR). The physical characteristics of the collimators are described in Table 2. Both LEHR and ELEGP, with their appropriate combination of hole diameter and length, are theoretically the best options for obtaining high resolution with low-energy photon emitters such as ^161^Tb. However, ^161^Tb also emits higher gamma photons, with energies up to 550 keV. The intensities of these photons are low, but some of them will penetrate the thin septa of the ELEGP and LEHR collimators. As a result, we also included the MEGP, with its thicker septa, to reduce the penetration effect of the high-energy photons. SPECT acquisitions were reconstructed using three ordered subset expectation maximization (OSEM) reconstruction projection models, namely, (1) attenuation-corrected (A-OSEM), (2) attenuation-corrected and scatter-corrected (AS-OSEM), and (3) attenuation-corrected, scatter-corrected, and detector-collimator response-corrected (ASC-OSEM). The latter was performed by MC simulation using the Sahlgrenska Academy Reconstruction code (SARec) [24]. The triple-energy window (TEW) method was used for scatter correction for EM1, while the dual-energy window (DEW) was used for EM2 in the AS-OSEM reconstruction. The A-OSEM and AS-OSEM reconstructions were performed at the clinical work station (Xeleris, GE). ASC-OSEM was implemented at the PhONSAi (the medical Physics, Oncology and Nuclear medicine research image platform at Sahlgrenska Academy) research station. When using SARec, the scattering in the collimator was modeled with a photon-scattering kernel. This kernel was determined from line source measurements using a triple-line phantom, as described earlier [24]. In this study, we also added a model of the spatial resolution in the backprojection, which was not included in our previous study [24]. The backprojection applies narrow beam attenuation, i.e., it does not include scattering. Analysis of the number of subsets and iteration revealed that 6 iterations and 10 subsets generated convergence for all spheres. As a result, this number of iterations and subsets was subsequently used for all reconstructions.

**Table 2:** Physical characteristics of LEHR, ELEGP, and MEGP collimators with hexagonal hole shapes

Collimator	Hole diameter [mm]	Septal thickness [mm]	Hole length [mm]
LEHR	1.5	0.2	35
ELEGP	2.5	0.5	40
MEGP	3.0	1.05	58

### Production of ^161^Tb

No-carrier-added (n.c.a.) ^161^Tb was produced at the spallation-induced neutron source (SINQ) at Paul Scherrer Institute (PSI; Villigen-PSI, Switzerland), at the high-flux reactor of the Institut Laue-Langevin (ILL; Grenoble, France) or at the SAFARI-1 reactor (Pelindaba, South Africa) with various masses of enriched ^160^Gd targets. ^161^Tb separation from the target material was carried out at PSI using cation exchange chromatography, followed by extraction chromatography, as previously reported [26]. Three separate shipments of approximately 2 GBq ^161^Tb each were transported from PSI to Sahlgrenska University Hospital. Diethylenetriaminepentaacetic acid (DTPA) was added to the aqueous solution of ^161^Tb to prevent adsorption of the activity to the plastic material of the phantom. It was observed that non-complexed ^161^TbCl_3_ (< 90% complexed by DTPA) adsorbed to plastic.

### Energy calibration, uniformity measurements, and sensitivity measurements

The γ-camera was energy calibrated, according to clinical routine, by placing a syringe containing ^99m^Tc at a distance far from the detector surface to generate a uniform flux. The calibration was performed intrinsically, i.e., without collimation.

Due to ring artifacts that can occur in SPECT images, a uniformity map was created using ^161^Tb. Artifacts may appear in SPECT images due to non-uniformity in the response over the detector surface [27]. Such artifacts may be reduced by acquiring an image of a uniform flood source and weighing the response in each pixel, such that the signal is uniform across all pixels. Uniformity correction maps can be either intrinsic, which consider the detector head uniformities, or extrinsic, which also incorporate the collimator [27].

A flood-source phantom, filled with an aqueous solution of ^161^Tb-DTPA, was placed between the detectors at approximately 5 cm from each detector surface. A matrix size of 256 × 256 was used and a total number of 10^8^ counts for the EM1 and EM2 maps were acquired separately. Subsequently, a Jaszczak phantom (described in more detail below) was imaged with and without uniformity correction, to assess the uniformity correction effect. Uniformity correction maps were created for all three collimators and applied to the subsequent Jaszczak phantom measurements.

Sensitivity measurements were carried out using a flat, circular, plastic phantom with a diameter of 11 cm, according to local clinical routine. A planar image was collected with data from detector 1 (on the table) and detector 2 (situated under the table). Another image was recorded, using the abovementioned setup, but swapping the two detectors. The detectors captured data for 15 min for each image produced. Sensitivity was calculated, per MBq, as the average of the two geometric mean counts from the images using both detector settings. Sensitivity measurements were only performed for the LEHR collimator, as the use of this collimator produced superior image quality.

### Phantom measurements

The Jaszczak SPECT imaging phantom was used for image quality assessment, as well as for the evaluation of imaging parameters. The phantom is cylindrical and contains six hollow spheres, with inner diameters of 10, 12, 16, 20, 25, and 31 mm, respectively. The phantom and spheres were filled with an aqueous solution having 92 and 920 kBq/mL of ^161^Tb-DTPA, respectively. The total SPECT measuring time, with 120 projections (matrix size of 128x128 with pixel sizes of 4.42 mm), was 3.3 h for the first imaging session. The long measuring time was performed for obtaining low noise levels, a prerequisite for detection of SPECT artifacts. The measuring time also generated similar counting statistics as might be obtained with a clinical SPECT protocol, with 120 projections and 30-s acquisition time of the kidney (mean counts in phantom) and tumors (mean counts in spheres). These estimates are based on ^177^Lu-DOTATATE data therapies at our clinic (data not shown), i.e., about 8 GBq activity. Later imaging sessions were prolonged to compensate for the ^161^Tb decay (*T*_1/2_ = 6.89 d). The CT images used in the SPECT/CT reconstructions were acquired using a 140-kV tube voltage, 2.5 mA, and a rotation speed of 2.6 rpm. The matrix size was 512 × 512, with a pixel size of 0.98 mm and a slice thickness of 5 mm. The vendor-implemented adaptive statistical iterative reconstruction (ASIP) was used for the generation of the CT images.

In order to compare quantitative image quality comparison between the different SPECT reconstructions, recovery coefficients (RC), and signal-to-noise ratios (SNR) were calculated.

1 $$ \mathrm{RC}=\frac{C_{\mathrm{M}}}{C_{\mathrm{A}}} $$

where *C*_M_ and *C*_A_ are the measured mean activity concentration and the actual activity concentration in the sphere of interest, respectively. The *C*_M_ is equal to the mean number of counts in a spherical VOI divided by the camera calibration factor Q. This factor was determined for each combination of collimator, energy window, and reconstruction method by:

2 $$ Q=\frac{N_{\mathrm{VOI}}}{A_{\mathrm{VOI}}} $$

where *N*_VOI_ is the counts measured in a spherical VOI of 400 cm^3^ placed in the Jaszczak phantom and *A*_VOI_ is the activity in the sphere, i.e., the activity concentration (92 kBq/mL) multiplied by the VOI volume. The initial activity concentration of 92 kBq/mL was decay-corrected to the time of gamma camera measurement.

The SNR was calculated according to:

3 $$ \mathrm{SNR}=\frac{N_{\mathrm{s}}-{N}_{\mathrm{B}}}{\sigma_{\mathrm{B}}} $$

where *N*_S_ is the mean count concentration in the sphere of interest and *N*_B_ the mean count concentration in a background VOI (VOI_B_) of 400 cm^3^. The VOI_B_ was placed at the opposite side of the spheres in the Jaszczak phantom, to ensure resolution-induced spill-over from the activity in the spheres was avoided. *σ*_B_ is the standard deviation of the mean count concentration in 20 VOIs, which is equal to the size of the sphere of interest. These spheres were located at different positions in the background region, as described above. In addition, the relative noise level, coefficient of variation (COV), was calculated as the standard deviation divided by the mean background count concentration:

4 $$ \mathrm{COV}=\frac{\sigma_{\mathrm{B}}}{N_{\mathrm{B}}} $$

The RC, SNR, and COV calculations were performed separately for images reconstructed from EM1 and EM2 data. ^161^Tb images were visually compared with ^177^Lu images and the image resolution determined.

Non-complexed ^161^TbCl_3_ had a tendency to adsorb to the plastic walls of the phantoms. This phenomenon enabled visualization of the different resolutions of the SPECT reconstruction methods. A lung phantom with spherical hollowed inserts was filled with an aqueous solution of ^161^Tb. After about 1 h, the solution was removed and the phantom washed twice with water and, finally, refilled with water. The phantom was scanned using SPECT and the three reconstruction methods described above were applied.

### Resolution analysis of ^161^Tb and ^177^Lu SPECT images

The image resolution of the measured radioactive (“hot”) spheres was determined from matched-filter analysis [28]. Digital versions of the spheres, with inner and outer diameters given by the manufacturer, were created and manually positioned over the respective spheres in the SPECT image. The spheres were filled with the same activity concentrations as used in the experimental setup, i.e., the “hot” sphere-to-background ratio was set to 10. Thereafter, a convolution with a 3D-Gaussian filter was applied to simulate the MC-SPECT resolution. The root mean square error (RMSE) between the voxels in the filtered digital spheres and the experimental spheres were calculated. This was repeated for different standard deviations (SD) in the Gaussian filter; SD from 2 to 12 mm, in 0.1-mm increments, was used. The SD with the smallest RMSE was used to calculate the matched-filter resolution; full width at half maximum (FWHM) was equal to 2.355*SD. This analysis was performed for all six spheres in the Jaszczak phantom, and the mean value was used as the resolution. The matched-filter analysis was also applied to previous MC-SPECT images of the Jaszczak phantom when filled with ^177^Lu [24]. Statistically significant differences in resolution between ^161^Tb and ^177^Lu imaging were determined with Student’s *t* test. *P* < 0.05 was considered statistically significant.

## Results

### Energy window setting and uniformity corrections

**Fig. 1:**
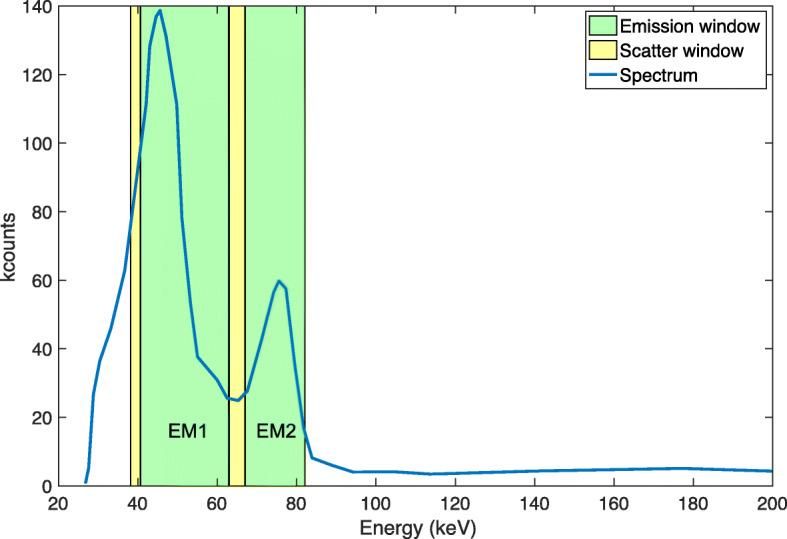
Spectrum from the γ-camera and the photopeak energy windows (green marked) and scatter windows (yellow marked)

The energy spectrum from the γ-camera demonstrated the problem of obtaining well-separated energy peaks for ^161^Tb (Fig. 1). The low-energy emission window (EM1) was around the main emission peak of 48.9 keV and had its lower and upper limit set at 40.7 and 62.9 keV, respectively. The upper limit was set to the lowest intensity level between the two main peaks 40.7 and 74.6 keV. The high-energy emission window (EM2) was focused on the main emission peak of 74.6 ± 10% keV. Two scatter windows were added to the configuration (Fig. 1; Table 3).

**Table 3:** Energy windows used for γ-camera imaging

Energy window	Lower limit [keV]	Center [keV]	Upper limit [keV]
SC1	38.2	39.4	40.6
EM1	40.7	48.9	62.9
SC2	63.0	65.0	67.0
EM2	67.1	74.6	82.1

**Fig. 2:**
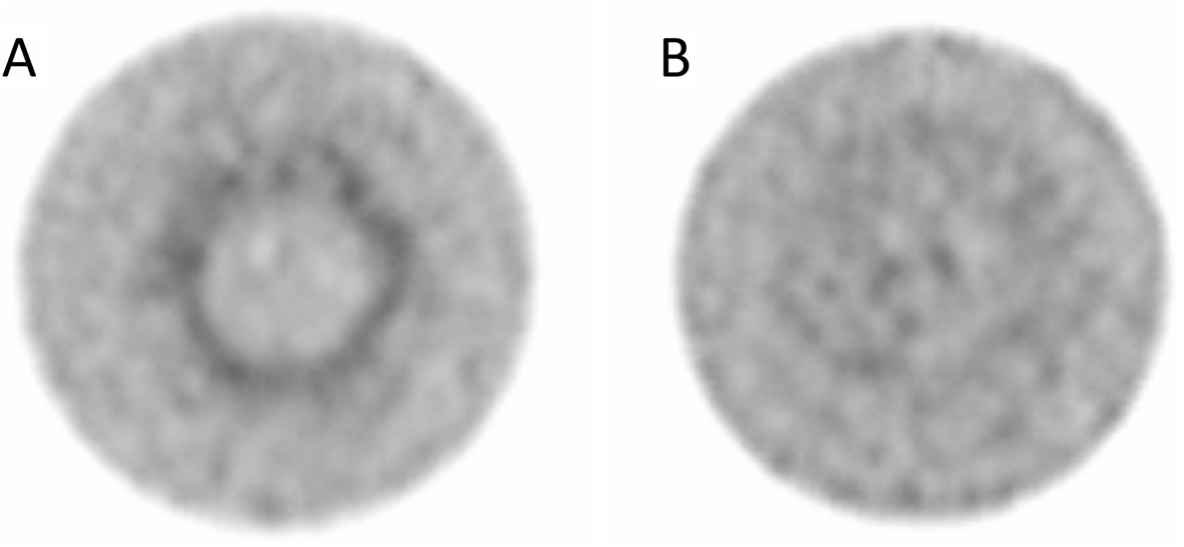
Intrinsic-corrected SPECT images, for EM2, of the Jaszczak phantom with a ring artifact (**a**) and extrinsic uniformity-corrected SPECT images with no visual ring artifact (**b**). The color scale is normalized to each image separately

The uniformity of the planar images of the flood-source phantom was deemed acceptable, but ring artifacts were present in the Jaszczak phantom after reconstruction (Fig. 2). The artifacts in the EM2 images were eliminated by applying extrinsic uniformity correction, while the artifacts seen on the EM1 images were reduced when applying the correction, but not removed entirely.

### SPECT reconstructions

**Fig. 3:**
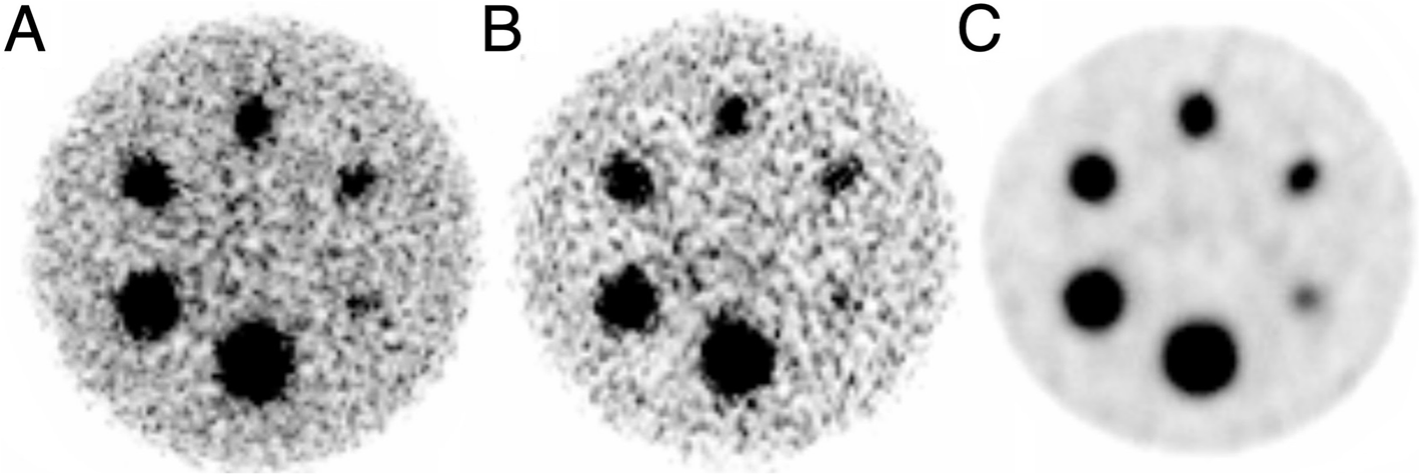
The Jaszczak phantom imaged using the LEHR collimator and reconstructed with AC-OSEM (**a**), ACSC-OSEM (**b**), and MC-OSEM (**c**) for EM2. The color scale is normalized to each image separately

**Fig. 4:**
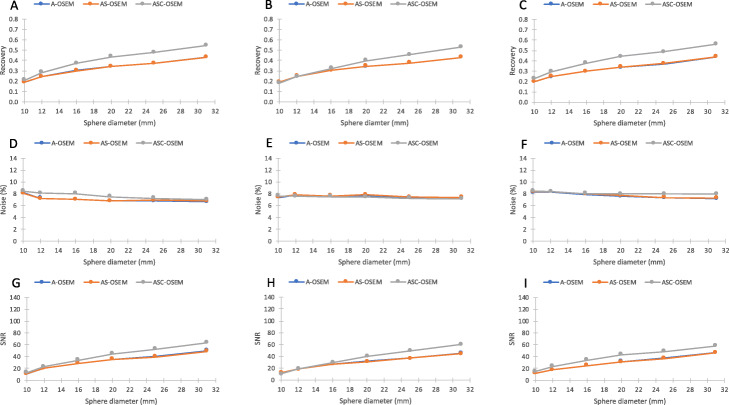
Recovery (**a**–**c**), relative noise (**d**–**f**), and signal-to-noise ratio (**g**–**i**) as a function of hot sphere diameter for images reconstructed using EM1 data. The data for the LEHR, ELEGP, and MEGP collimators are in first column (**a**, **d**, **g**), second column (**b**, **e**, **h**), and third column (**c**, **f**, **i**), respectively. Observe that the data for A-OSEM and AS-OSEM is frequently undistinguishable

**Fig. 5:**
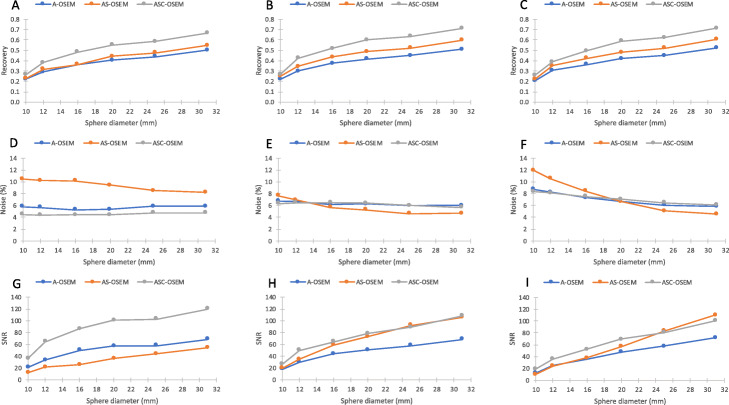
Recovery (**a**–**c**), relative noise (**d**–**f**), and signal-to-noise ratio (**g**–**i**) as a function of hot sphere diameter for images reconstructed using EM2 data. The data for the LEHR, ELEGP, and MEGP collimators are in the first column (**a**, **d**, **g**), second column (**b**, **e**, **h**), and third column (**c**, **f**, **i**), respectively

**Fig. 6:**
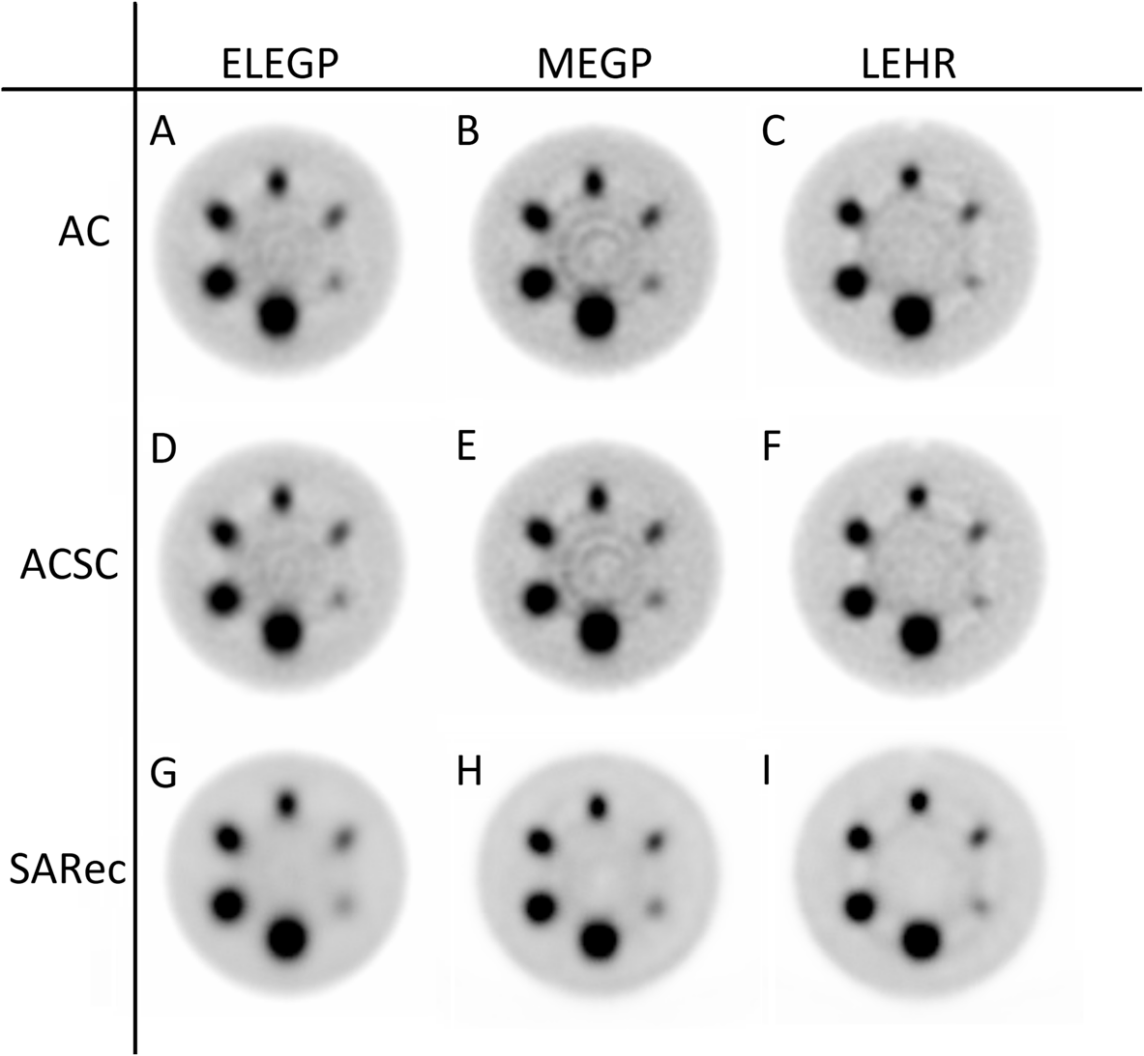
The Jaszczak phantom imaged with ELEGP (A/D/G), MEGP (B/E/H), and LEHR (C/F/I) collimators and reconstructed with A-OSEM (A/B/C), AS-OSEM (D/E/F), and ASC-OSEM (G/H/I), respectively, for EM1. A-OSEM and AC-OSEM images were postfiltered using a Butterworth filter of order 2 and cutoff frequency 0.08 cycles/mm. The color scale is normalized to each image separately

**Fig. 7:**
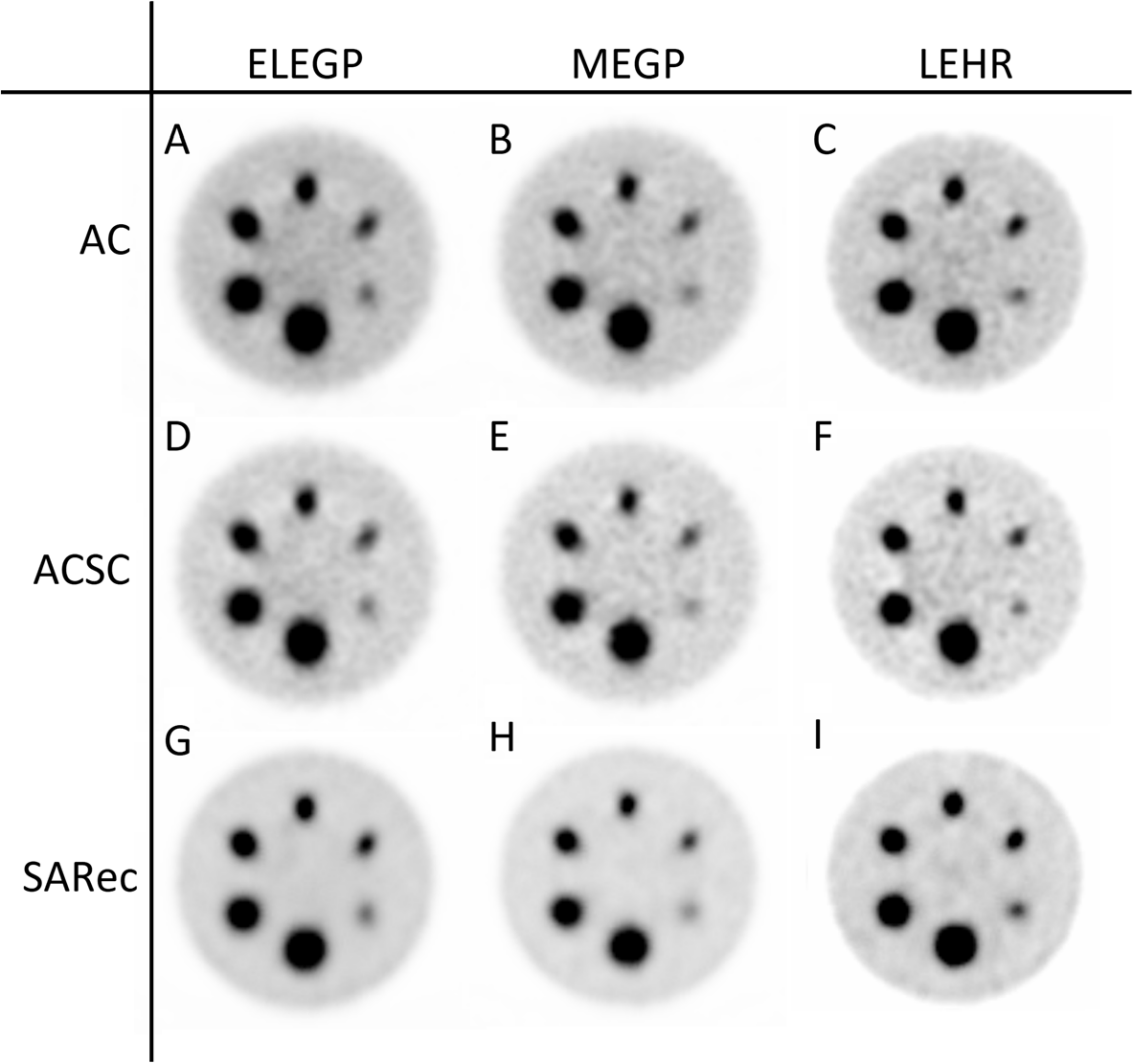
The Jaszczak phantom imaged with ELEGP (A/D/G), MEGP (B/E/H), and LEHR (C/F/I) collimators and reconstructed with A-OSEM (A/B/C), AS-OSEM (D/E/F), and ASC-OSEM (G/H/I), respectively, for EM2. A-OSEM and AS-OSEM images were postfiltered using a Butterworth filter of order 2 and cutoff frequency 0.08 cycles/mm. The color scale is normalized to each image separately

The SPECT image reconstruction with A-OSEM and AC-OSEM, respectively, resulted in images with high noise levels (Fig. 3). The noise was substantially reduced and the recovery improved when ASC-OSEM was applied. The SPECT images reconstructed with EM2 data had better recovery and SNR compared to the reconstruction with EM1 data (Figs. 4, 5, 6, and 7). Analysis of the EM1 data indicated that the window-based scatter correction had no effect on the quantitative SPECT images parameters, while for EM2 data the recovery and SNR was improved (Fig. 4). The ASC-OSEM-reconstructed image provided the highest recovery coefficients for all the “hot” spheres, which was similar for all collimators (Figs. 4 and 5). When ASC-OSEM reconstruction was performed, the largest sphere had RC ranged from 0.67–0.71, while for A-OSEM and AS-OSEM reconstructions the RC was 0.50–0.51 and 0.55–60, respectively. When ASC-OSEM reconstruction was utilized, the SNR was highest for the LEHR collimator and lowest for the MEGP collimator.

By filtering the A-OSEM and AS-OSEM reconstructed images with a Butterworth filter of order 2 and cutoff frequency 0.08 cycles/mm, the image noise was reduced and the visual impression improved (Fig. 7). When comparing the impact of the collimators on image quality of the filtered images, it was visually determined that the LEHR images had higher resolution, had better rendering of the spheres, and showed more defined edges of the spheres than the ELEGP and MEGP images. Figure 6 demonstrates the ring artifacts obtained, despite extrinsic uniformity correction, for SPECT images reconstructed with EM1 data.

**Fig. 8:**
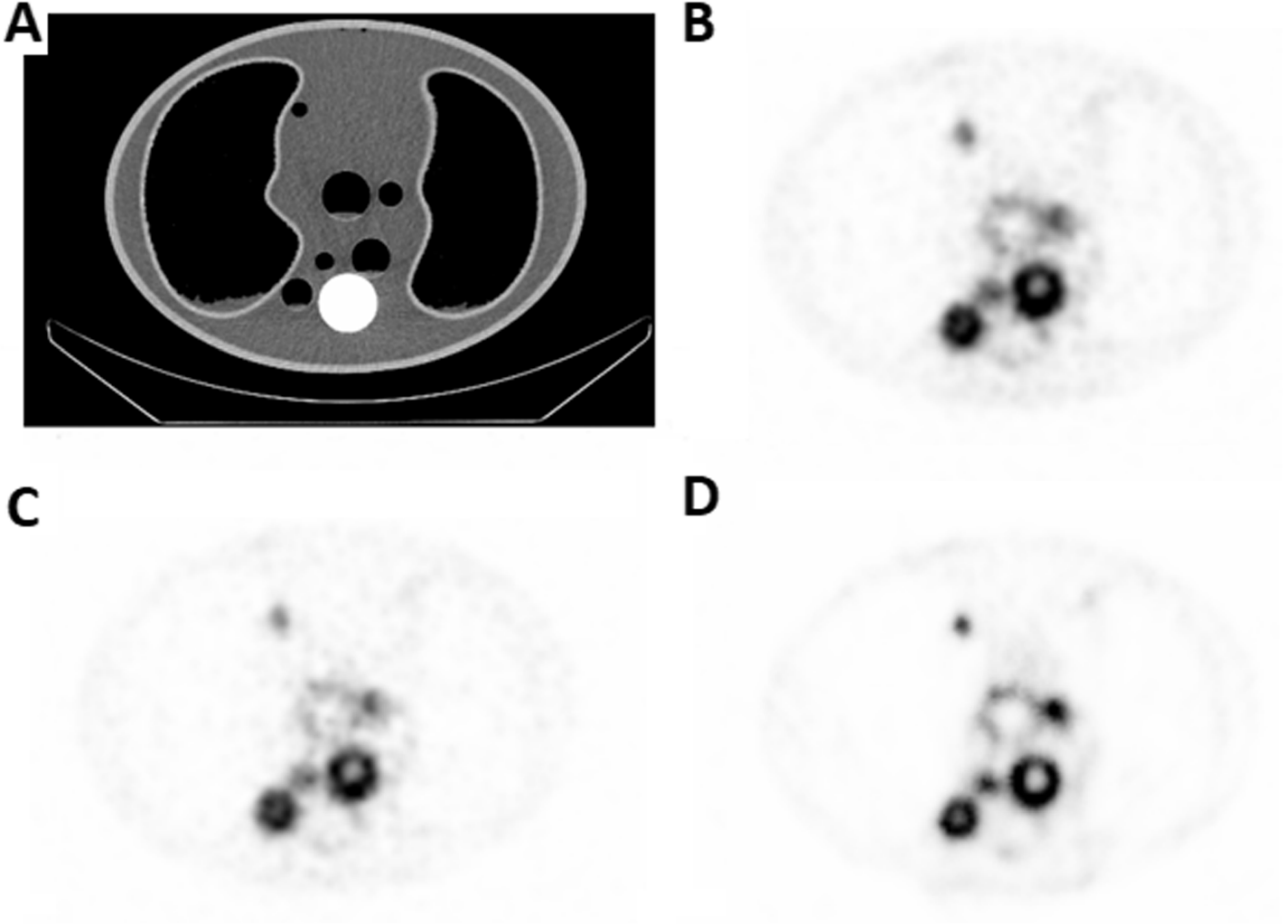
The lung phantom with ^161^Tb attached to the plastic walls of the different inserts. CT images of the lung phantom (**a**). SPECT images reconstructed with A-OSEM (**b**), AS-OSEM (**c**), and ASC-OSEM (**d**), respectively. The color scale is normalized to each image separately.

Non-complexed ^161^Tb had a tendency to adsorb to the plastic walls of the phantoms. This situation enabled the visualization of different resolutions of the SPECT reconstruction methods (Fig. 8). The ASC-OSEM images (Fig. 8d), with its high resolution, visualized the ^161^Tb adsorption to the plastic walls better than the A-OSEM and AS-OSEM images (Fig. 8b, c, respectively).

### Resolution and sensitivity analysis of ^161^Tb and ^177^Lu SPECT images

**Fig. 9:**
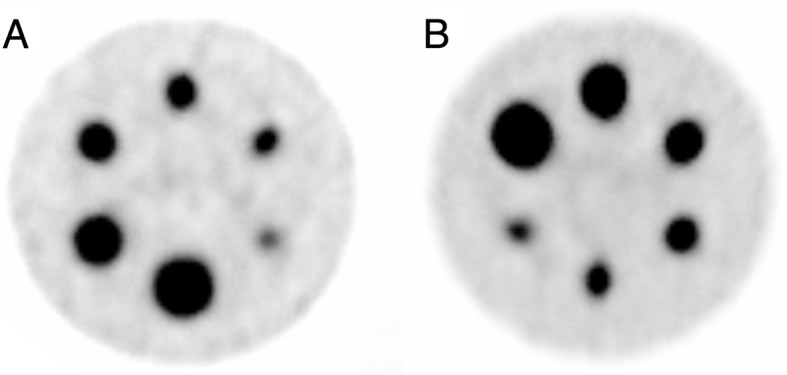
ASC-OSEM reconstructed SPECT images of ^161^Tb (**a**) and ^177^Lu (**b**) with hot-sphere-to-background ratios of 10 and 25, respectively. The color scale is normalized to each image separately

The hot spheres of the Jaszczak phantom images filled with ^161^Tb and ^177^Lu, respectively, were compared (Fig. 9). The ^177^Lu data were obtained from Ryden et al., where the “hot”-sphere-to-background ratio was equal to 25 [24], while in the present study the “hot”-sphere-to-background ratio for ^161^Tb was 10. When applying matched-filter analysis on these images, ^161^Tb resulted in an image with a statistically significantly higher resolution (8.4 ± 0.7 mm) than the resolution obtained with ^177^Lu (10.4 ± 0.6 mm). The sensitivity of planar images with EM2 and LEHR collimator was determined to be 7.41 cps/MBq for ^161^Tb, while the sensitivity was calculated to be 8.46 cps/MBq for ^177^Lu, for a 20% energy window over the 208 keV peak and a MEGP collimator.

## Discussion

Theoretical dose calculations and experimental data both suggest that using ^161^Tb for the treatment of metastatic disease would be more favorable than ^177^Lu [3, 15, 16]. The reason for these results is the higher emission rate of low-energy internal conversion and Auger electrons of ^161^Tb (~ 12 e^−^, ~ 36 keV/decay) as compared to ^177^Lu (~ 1 e^−^, ~ 1.0 keV/decay). Therefore, ^161^Tb will deliver a higher total absorbed dose to micro-metastases than ^177^Lu [3, 4]. In view of ^161^Tb clinical application, it will be of interest to perform SPECT imaging for dosimetry of critical organs and tumors. In this study, we demonstrated that the use of ^161^Tb can produce high image quality, which provides SPECT images of higher resolution as compared to the clinically-used ^177^Lu.

Although the 25.7 keV γ-emission from ^161^Tb has the highest photon yield, this energy could not be employed since the γ-camera used for this study could not measure energies lower than 33 keV. As a result, a test was performed using an energy window centered on the energy of the photon with the next highest yield, namely, the 48.9 keV peak. It is still low energy to measure for a γ-camera, however. The signal using this window will be substantially degraded by the exceptionally poor energy resolution of such low-energy photons imaged with a γ-camera, and will also be influenced by scattered photons from the 74.6 keV peak. A scatter correction is, therefore, most challenging to perform for this low-energy window. This study demonstrated poorer image quality for the 48.9 keV peak compared to the 74.6 keV peak. From this perspective, it is more adequate to use the γ-emission with the highest energy. Furthermore, the attenuation is more affected by energy variations in the low-energy range than in the higher range. Since there are several X-ray emissions in the low-energy window (EM1), as well as scattered photons of various energies, the variation will be large and attenuation correction challenging.

Pronounced ring artifacts in the SPECT images were found, despite approved uniformity calibration of the planar images. Consequently, the clinical standardized uniformity map was not accurate enough to ensure the absence of ring artifacts in ^161^Tb SPECT images. When applying intrinsic uniformity correction, the ring artifacts were reduced, but still present. This was unfavorable, since the methodology for this correction is easy to apply. The use of extrinsic uniformity correction, however, reduced all visible ring artifacts for the SPECT images reconstructed with data from EM2. When SPECT images were reconstructed with EM1 data the artifacts were reduced, but still present. At this stage, it can only be speculated that the ring artifacts were not removed for the lowest energy window due to the fact that attenuation is increased with decreased photon energy. As a result, imperfections in the collimator will influence the uniformity more for EM1 than EM2. The cause of ring artifacts has been studied to some degree [27]; however, the above speculation has, to our knowledge, not been carefully investigated. It is, thus, strongly encouraged to carry out extrinsic uniformity correction before imaging with ^161^Tb.

The above considerations, as well as the different visual appearance of the images reconstructed using EM1 and EM2, led to the conclusion that the application of EM2 is best suited for SPECT imaging using ^161^Tb.

All three collimators under investigation seemed to generate acceptable SPECT images with regard to the qualitative parameters used. However, when taking visual inspection into consideration, the LEHR collimator was considered the best option, while with ASC-OSEM reconstruction it obtained the highest SNR values. The LEHR collimator should provide the best image resolution and recovery, due to its small holes and thin septa, but the recovery was similar for all collimators, which indicates that some photon penetration was obtained in the LEHR collimator. No streak artifact was observed in the projections, but this issue needs to be further explored. Nevertheless, the ^161^Tb-based SPECT image had a higher resolution using the LEHR collimator than the image obtained when using ^177^Lu (which uses the MEGP collimator for SPECT imaging). This was presumably due to the lower photon energies emitted by ^161^Tb over ^177^Lu.

Three different OSEM algorithms were utilized to analyze the effects of attenuation and scatter correction, as well as resolution recovery correction, on the image quality using ^161^Tb. When using A-OSEM, it was feasible to obtain an acceptable SPECT image quality. The application of scatter correction improved the recovery slightly, but it also introduced increased high-frequency noise. In contrast, both the recovery and SNR were clearly improved when ASC-OSEM was used. One reason for these improvements is probably due to the lower noise level and higher contrast for MC-based scatter correction, as demonstrated for ^99m^Tc-SPECT [29]. These authors also demonstrated that MC-based scatter correction is less sensitive to anatomical variations, making it theoretically favorable for clinical SPECT imaging.

The MC code SARec was developed to optimize parallelization of simulated photons emitted from each voxel. Each ASC-OSEM reconstruction took only a few minutes to complete. When performing the initial simulation using SARec, the forward projection in the ASC-OSEM reconstruction was simulated, which resulted in clearly improved image resolution [24]. Since the backprojection was not simulated in the ASC-OSEM reconstruction, the noise level was in parity with AC-OSEM. When applying a code with modeling of the spatial resolution in the backprojection step, SNR clearly improved. As a result, no post-filtering was applied to the ASC-OSEM reconstructions, which would have resulted in lower spatial resolution.

When using ASC-OSEM, both the correction of scattering and depth-dependent resolution are taken into account. Various vendors take these effects into account by different approximation methodologies in their reconstruction algorithms, which is only partly divulged to the end-user. In this work, no specific vendor methodology to handle the depth-dependent resolution correction was investigated. It was demonstrated, however, that ASC-OSEM clearly improved SPECT image quality compared to A-OSEM and AS-OSEM. Similar results are anticipated when applying vendor-specific depth-dependent resolution corrections. Nevertheless, the clinical value of these results had to be proven. Hippeläinen et al. demonstrated, in a small retrospective study (10 patients), that inclusion of MC-OSEM-based scatter correction to attenuated, and collimator-detector corrected, ^177^Lu-SPECT images increased the recovery, thereby, a 19–25% higher mean absorbed kidney dose was obtained [30]. However, since full recovery is still not obtained for larger organ sizes, such as kidneys, it is still challenging to compensate for the remaining partial volume effect. Despite the superior properties of MC-based reconstruction, or other attenuation, scatter, and collimator-detector response correction methods, it still has to prove its value over A-OSEM in clinical dosimetry protocols.

## Conclusion

It is feasible to perform SPECT imaging using therapeutic quantities of ^161^Tb and obtain good image quality, with an energy window centered at 74.6 keV (67.1–82.1 keV). The LEHR collimator is a good choice of collimator for high resolution of the resultant images. Monte Carlo-based OSEM generated higher image quality than A-OSEM and AS-OSEM reconstructions. Since the sensitivity between ^177^Lu and ^161^Tb is comparable, it can be concluded that dosimetry using ^161^Tb-labeled radiopharmaceuticals in a clinical setting is feasible.

## Data Availability

The datasets used and/or analyzed during the current study are available from the corresponding author on reasonable request.
